# The natural defense system and the normative self model

**DOI:** 10.12688/f1000research.8518.1

**Published:** 2016-05-03

**Authors:** Philippe Kourilsky

**Affiliations:** 1 Department of Immunology, Institut Pasteur, Paris, France; 2 Center for Interdisciplinary Research in Biology, CNRS/UMR 7241 - INSERM U1050, Collège de France, Paris, France

**Keywords:** systems biology, complexity, physiology, robustness, immune system, quality control, network, self non self discrimination

## Abstract

Infectious agents are not the only agressors, and the immune system is not the sole defender of the organism. In an enlarged perspective, the ‘normative self model’ postulates that a ‘natural defense system’ protects man and other complex organisms against the environmental and internal hazards of life, including infections and cancers. It involves multiple error detection and correction mechanisms that confer robustness to the body at all levels of its organization. According to the model, the self relies on a set of physiological norms, and NONself (meaning : Non Obedient to the Norms of the self) is anything ‘off-norms’. The natural defense system comprises a set of ‘civil defenses’ (to which all cells in organs and tissues contribute), and a ‘professional army ‘, made of a smaller set of mobile cells. Mobile and non mobile cells differ in their tuning abilities. Tuning extends the recognition capabilities of NONself by the mobile cells, which increase their defensive function. To prevent them to drift, which would compromise self/NONself discrimination, the more plastic mobile cells need to periodically refer to the more stable non mobile cells to keep within physiological standards.

## Introduction

Living organisms are protected by their immune system from infections. They are also threatened by the many errors which occur in their body. The ‘normative self model’ proposes that all abnormalities and disorders are taken care of by a unique system of natural defenses, which includes but exceeds the immune system ^1^. After describing its composition and global architecture, I will show how it may protect the physiological self without attacking it. Overall, the theory, (which makes use of the tuning concept developed by Grossman and Paul ^2^) is consistent and may help in understanding various physiological and pathological situations.

## The hazards against which the organism must be protected

### Enemies from the outside and enemies from the inside

External enemies come from the environment and mostly include infectious agents. Internal enemies, such as cancer cells, originate from the *numerous mistakes* which occur continually, within the body. They are often underestimated because most are corrected by quality control mechanisms. They include: errors in DNA replication, epigenetic alterations, incorrect transcription and splicing, errors in the synthesis ^3^ and modification ^4^ of proteins retrotransposon jumping ^5^, unproper cellular migration, illegitimate cell pairing, organ dysfunction (such as, extracystoles in heart beats), etc.

### Quality control and error correction mechanisms

Most biological mechanisms involve a series of steps. Each has a small, but significant probability of error. Biological processes may be seen as a succession of trials and errors which converge towards the correct biological solution, under conditions which have been algorithmically formalized ^6^. Each step has a certain yield, and a certain specificity. The combination of moderately specific events may produce an exquisitely specific output (though the final result may occasionally be incorrect). Such processes generate waste which must be recycled or eliminated (as in DNA replication and protein synthesis).

Error rates vary widely. The more steps a biological process involves, the more mistakes it is likely to make, even after error correction. Thus, the frequency of misincorporation of a nucleotide into replicating DNA is about 10 ^-9^; that of a wrong aminoacid into a protein (which involves many more steps) is higher than 10 ^-5^. Error rates would be higher, and unbearable for the organism, if it were not equipped with multiple quality control mechanisms. However, too many would consume too much time and/or energy. A ‘too close to perfect’ organism would not be competitive enough to survive. Therefore, mistakes are inevitable. The observed rates result from highly selected evolutionary trade-offs.

At a higher degree of organization, quality control mechanisms are found in the immune system, since (i) its own processes, (such as T lymphocyte selection) are quality controlled; (ii) it is itself a major quality control device, since it cures many unnoticed infections (revealed by antibodies in the serum of healthy people). It is likely that the same happens in at least certain cancers (as suggested, in particular, by the early dissemination of metastatic cells, which are kept silent by immune cells ^7^). By extension, the natural defense system may cure many other adverse events (micro-bleeding, minor cardiovascular accidents, etc) ^8^. However, this logical assumption awaits further experimental support, because error corrections are difficult to observe, unless sequellas remain after repair.

## The architecture of the natural defense system

### The immune system and beyond

It is now accepted that the immune system does not only fight infectious agents ^9^, it also intervenes in cancer ^10^, so much so that several classical chemotherapeutic anti-tumor drugs (such as Temozolomide) have been shown to activate the immune system ^11^. The belief that the adaptive system appeared once in evolution has been challenged by the finding that lamprey and hagfish have evolved an adaptive system which relies on molecules with LRR (leucin rich repeats) motifs, instead of the usual immunoglobulin fold ^12^. Moreover, it was discovered recently that (like plants) certain bacterial species have evolved micro-RNA based adaptive immunity to destroy the genomes of infecting bacteriophages ^13^. Finally, the borders of the immune system have been expanded, due to its functional relationships with the gut microbiota ^14^ and the nervous system ^15^. Thus, the limits of the immune system have changed several times.

**Figure 1.**
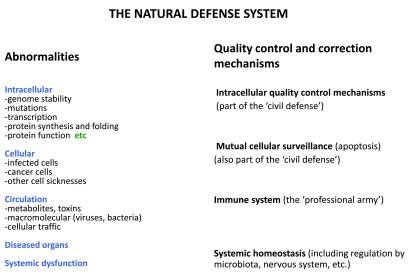
The natural defense system includes and exceeds the immune system. Aberrations and mistakes occur at all levels of the organism, and are listed on the left side. The major categories of quality control mechanisms are shown on the right. The immune system, (which makes up most of the ‘professional army’), covers one part. Intracellular quality controls, and mutual cellular surveillance (often by apoptosis) belong to ‘civil defenses’ which are individually exerted by all cells of the body (including those composing the professional army). Roads to diseases, and particularly to cancers rely on multiple aberrations that are not corrected by several of these quality controls.

The proposed ‘natural defense system’ considers everything that needs to be fixed in the organism. For example, neither clotting factors, nor piwi ^16^, are considered part of the immune system. However, they do appear as *bona fide* defenses when one considers the potential damages generated by wounds or human genome destabilization by retrotransposons (including cancers ^17^). Pain is a major warning system. Microbiota (in the gut and elsewhere), and mucus synthesis in the lung also help prevent infections. All contribute to defend the body against the hazards of life ( Figure 1) and belong to the natural defense system. Because of its fuzzy borders, its size is hard to estimate. It must be significantly larger than the immune system, (at least a few % of human genes ^18^). Arguably, adding up ‘cancer’ genes, plus some genes involved in the nervous, neuroendocrine and coagulation systems, might total 20% or more.

### A professional army and civil defenses make up the natural defense system

From now on, my use of military metaphores (common in immunology) should not obscure the preventive role of the natural defense system, which (more like a doctor than a soldier) solves many problems before they become pathological.

The body contains a vast majority of immobile cells, of which organs and tissues are made, and a minority of mobile cells, mostly found in blood and lymph. Others are motile; they reside in tissues and start moving upon stimulation (for simplicity, I will refer to them as ‘mobile’). Mobile cells exert a key defensive role, by moving to problematic site, (cf immune inflammation), where some proliferate, and/or function in destruction (eg cytolysis), or repair (clotting and healing). The ‘professional army’ of the organism, mostly recruited amongst these mobile cells, complements the ‘civil defenses’ provided by all cells, mobile and non mobile (which, for example, make interferons upon viral infection). Civil defenses include a large variety of intracellular and systemic quality control mechanisms (such as those involved in DNA replication, metabolism and temperature regulation). I will not discuss the idea that the human brain is a rupture innovation which allows man to manipulate its own natural defenses and invent new ones.

## The internal consistency of the natural defense system

### Shared features of the natural defense system

***Enemies from the outside and the inside are not fully distinguishable.*** Pathogens which penetrate inside the body are both recognized by their ‘foreign’ molecular motifs (such as bacterial lipopolysaccharide LPS), and by the disorders which they cause. The latter (such as death by necrosis instead of apoptosis, which delivers strong signals to the immune system), may be similar in infection, cancer and other pathological situations, so that the associated abnormalities may be detected and corrected by the same mechanisms. For example, the CD1 non-classical class I MHC molecules check on lipid metabolism by presenting particular lipids and metabolites to specific T cells, which in turn release cytokines in a variety of settings ^19, 20^. Reciprocally, tumors, as infecting pathogens, elicit specific antibodies and cytolytic T cells. Immune effector mechanisms (particularly macrophages) intervene in a variety of healing situations. Skin wounds call on coagulation devices as well as on immune cells and cytokines. Neuromediators are often linked to inflammation, chronobiology modulates defenses against infections, neuro-immuno-psychology starts making molecular sense ^21^, and so on. Therefore, defense mechanisms often operate across various non physiological situations.

***The continuum between the biology and the immunology of tumors.*** Tumor immunology has had its ups and downs, (immunology was absent from a seminal review on cancer written in 2000 ^22^), but there is now overwhelming evidence that the immune system fights tumors. Correlatively, tumor immunotherapy is gaining ground, for example with the Chimeric Antigen Receptors (CAR) approach ^23^. Of course, the immune system intervenes late, once several quality control mechanisms have failed. These include the intracellular control of mutations in pre-tumoral cells, and p53 related mechanisms which signal abnormalities, resulting in the apoptosis of the diseased cell, triggered either from within itself, or by neighbouring ones. It is only after a number of steps ^24^, including progressive modifications of the tumor microenvironment ^25^, that the immune system, with which it interacts ^26^, takes action. In my view, this continuum of anti-tumor devices is better accommodated under the label of ‘natural defenses’, rather than sliced into at best overlapping segments as usually done.

The natural defense system intervenes at different levels to maintain the integrity of molecules (nucleic acids and proteins), individual cells, increasingly large sets of cells, up to organs, systems, and the entire body. Cancers of genetic origin involve these various levels, starting with the prevention of mutations up to organs. Infections skip the first round of genetic controls. The repair of an accidental, external, wound mobilizes the later stages. Multiple entries and exits are thus plugged on a general backbone, which links molecules to body parts and the entire body.

***The set of natural defenses makes up a system.*** Natural defenses would not belong to a ‘system’ if they were a mere collection of disparate devices. Instead, they include many interconnected surveillance and correction mechanisms. In particular, apoptosis, although not usually presented as such, is a common quality control mechanism, which operates across the natural defense system. It is linked to the surveillance of many major intracellular failures; it is also related to mitochondria and to the utilization of energy ^27^. It is a radical correction mechanism, when it comes to suppress a cellular problem that could not be solved. It operates both intra- and inter- cellularly, since healthy cells may deliver an apoptosis signal to sick ones.

### Robustness is the conceptual cement of the natural defense system

***About complexity.*** Thanks to new technology and informatics, biological complexity can now be adressed more comprehensively, as illustrated by genome sequencing. However, as I discussed elsewhere ^28^, one should not confuse ‘ *systematic*’ and ‘ *systemic’* biology. The former documents complexity, without explaining it. As emphasized by S. Brenner ^29^, the massive acquisition of ‘big data’ does not (and cannot) suffice to solve major biological problems. Instead, complexity requires specific conceptual tools.

I now mention a few seminal papers. Engineers have dealt with complexity well before biologists. The notion of modularity was emphasized by Hartwell *et al.* (1999) ^30^. Csete and Doyle (2002) ^31^ have elaborated on a comparison between complex human artefacts (such as aircrafts) and biological systems. They showed that complexity in engineering and biology share the representation of objects by networks, the concept of an emergent property, and that of robustness. Later, Liu *et al.* (2011) ^32^ used control theory to convincingly suggest that living systems are particularly complex, since it appears necessary to control the vast majority of their nodes (about 80%) to master the evolution of the biological networks analyzed ( *versus* about 30% for the european electricity network).

***The definition of robustness.*** In the 1860’s, Claude Bernard defined homeostasis as the ability of a system to maintain a balanced functioning despite outside constraints. Hence the well known quote: *“The constancy of the internal environment is the condition for free and independent life: the mechanism that makes it possible is that which assured the maintenance, within the internal environment, of all the conditions necessary for the life of the elements”* ^33^. This concept strongly influenced cybernetics and engineering. Today, the notion of robustness (which some call ‘resilience’) adds to homeostasis, by equally considering the uncertainties associated with the « milieu intérieur » and the outside. Thus, Csete and Doyle (2002) ^32^ define robustness as “ *the preservation of particular characteristics of a system despite uncertainties in components or the environment”.* In a kind of pendulum’s swing, they make the point that it applies to biological systems. In my own words: “ *Robustness is a property that enables a complex system to keep on working, decently if not optimally, in spite of environmental hazards and internal failures”* (the term ‘ *decently*’ means that the system may go on working in a sub-optimal rather than optimal fashion).

***The natural defense system confers robustness to the organism.*** Thus, robustness in engineering matches the role here assigned to the natural defense system. In other terms, the function of the latter is to confer robustness to the organism. This statement is important, because it provides the natural defense system with a unified function which makes evolutionary sense. Robustness is likely to be a major driver of evolution ^34^ (at least within each species). In engineered systems, the space dedicated to robustness grows with time (the first aicrafts were much less robust than the current ones) and occupies more space. In living organisms, the number of « essential » genes ^35^ is relatively low. A ‘minimal’ bacterium needs a few hundred genes, while *E. coli* has about 4 000. Therefore, more than 3 000 might be dedicated to robustness, including adaptative capabilities (for instance, using lactose instead of glucose).

## The natural defense system is the guardian of physiology

### The physiological self

Thanks to its surveillance, correction and repair capabilities, the natural defense system is the guardian of *physiology*, physiology being, in its medical sense, opposed to pathology. The term is philosophically and practically imprinted with the notion of *normality*, which is a basis of medical thinking ^36^. The assessment of normality is heterogeneous in time, populations and cultures, without loosing its medical operational value.

When identifying and fighting external and internal hazards, the natural defense system must not damage ‘the physiological self’ that is, “ *the physiological organism at all stages of its life”*. This definition incorporates the dimension of time, since the body changes from childhood to adult and old age. The terms ‘physiology’ and ‘physiological’ refer to ‘ *norms’* and ‘ *normal’*, which themselves concern molecular, cellular and multicellular *structures* and *functions*, and their way of responding to the environment.

***The structural dimension.*** The physiological body is made of a vast number of structurally ‘normal’ molecules, cells and organs. Their catalogues are more and more comprehensive, though none is completed yet. New human genes, cell types, and even organs continue to be discovered. The gut microbiota is now considered a *bona fide* organ and the source of new metabolites ^37^. Combinations of cell surface sugars are still being explored ^38^. Thus, the composition and borders of the human body are still in question.

The numerous genetic polymorphisms further complicate the situation. For instance, major histocompatibility (MHC) class I molecules present a certain subset of self peptides to T cells. However, this ‘peptidic self’ ^39^ is almost unique to each person, because it is specified by the combination of MHC alleles borne by an individual. This feature partially accounts for MHC restriction and alloreactivity ^40^. So, there cannot be a unique physiological catalogue of self structures shared by all. Polymorphisms also blur the notion of ‘normality’. For example, it may be difficult to define a ‘normal’ gene crowded with hundreds of polymorphisms, (and even more a ‘normal’ genome) without refering to its ‘normal’ function.

***The functional dimension.*** After having adressed organic and systemic functions, physiology and pathology are now associated with cells and molecules. (for example, blood transaminases serve as an indicator of liver function). The genetic, epigenetic and environmental diversity, on top of functional fluctuations, broadens the standards of normality (cf. lymphocyte numbers and electrolyte concentration in a blood formula). The latter may reside in combinations of parameters, reflecting distinct states of biological networks. This is a trend in biomarkers research ^41^.

### The physiological self is self-assessed and characterized by a large set of norms

At any time in life, the system must be ‘aware’ of the physiological standards, and make use of them to detect and correct defects, without damaging what works. In other terms, *the physiological self is self-assessed.* This statement is neither paradoxical nor tautological. It refers to the general paradigm of *body development plans*, of which the physiological self is a part. As for any development plan, it is somehow rooted in the genome, while exceeding by far the simplistic interpretation of a direct genetic control over its development and implementation.

The large corpus of physiological norms, and the complementary one of structural and functional abnormalities, grow rapidly, thanks to sustained biomedical research. Beyond the morphological norms (much improved by the progress of imaging), many norms deal with the molecular and/or cellular components of organs or systems, reflecting the activity of cell types, and the nature of cellular interactions. Therefore, many physiological norms lie *in affinity and avidity constants* which rule the interactions between molecules, molecules and cells and/or between cells (through their surface molecules). They are also part of the body plan.

### The NON-self ( *Non Obedient to the Norms of the self*)

***Definition.*** What the natural defense system identifies and fights, therefore, is anything ‘off norms’, which I will refer to by the acronyme *“NON-self”*, meaning “ *Non obedient to the norms of the self*”. The NON-self is thus defined by default. It includes everything abnormal, either ‘foreign’, or self. Like the physiological self, the NON-self has a structural arm (a mutated DNA sequence, bacterial LPS, a misfolded self protein, molecular patterns or aggregates with an unusual geometry, etc), and a functional one (aberrant metabolism, organ dysfunction, etc). Note that a ‘non physiological’ feature is only *potentially* (rather than necessarily) pathological, since, most of the time, it is corrected by the natural defense system.

***NON-self is mostly inhabited by chance.*** Most adverse events which threaten the physiological self are fortuitous. Even if their probability of occurrence is modulated by the environment and the ‘milieu intérieur’, most hazards of life are not deterministic. Infections, wounds, deleterious mutations and other internal mistakes, happen by chance. Furthermore, the number of possible hazards is huge, and some (such as an infection by a newly evolved pathogen) are unpredictable. Therefore, *a major task of the natural defense system is to cope with chance*. This feature profoundly imprints the mechanisms of discrimination between self and NON-self, which themselves exploit chance (as illustrated in the immune system by the random recombination of antibody and TCR gene segments and by the Darwinian process of antibody maturation in germinal centers).

### The issue of a robust self/NON-self discrimination

NON-self is not ignored, but dealt with. Infectious agents and cancer cells trigger specific actions against them. Similarly, repair activities are focused on the abnormal zones. NON-self is defined by default, but it is actively recognized. The famous, and much studied, problem of self/non self discrimination by the immune system, has to be translated into an issue of (physiological) self/NON-self discrimination by the natural defense system.

Because it is essential for survival, the natural defense system is necessarily robust. The same holds for self/NON-self discrimination, which must include several, possibly redundant, mechanisms, and various quality control devices (as well documented in the immune system). Furthermore, its enemies have been selected to be robust. Most pathogens have evolved very sophisticated escape mechanisms, without which they would be harmless. Poliovirus, HIV-1 and others have concentrated in their small genomes an amazing number of firewalls against the immune system. Similarly, cancers develop out of complex and elaborate selective processes, which yield cells that are robust enough to defeat the natural defense system. It may be expected that serious heart strokes, vascular failures and other pathologies occur after a long series of repaired defects, but this needs to be substantiated.

## The normative self model

### Lessons from the immune system

***Previous theories of immune self/non self discrimination.*** The clonal deletion theory, first formulated in 1949 by Burnett, claimed that self-reactive immune cells are destroyed or inactivated. It was beautifully simple and dominated for decades, but did not account for the presence of numerous autoreactive B and T cells, and abundant self-reactive antibodies in blood and lymph. The resurrection of (suppressive) regulatory T cells ^42, 43^ added a well needed negative loop to explain major aspects of tolerance to self. The “danger theory” ^44^ claimed that self constituents, if dangerous, can trigger an immune response (while non dangerous, non self constituents will not). The strengths and weaknesses of this approach have been discussed ^45^. If clearly not generally applicable, it has emphasized the functional aspect of the physiological self (inasmuch as ‘danger’ is abnormal). The “discontinuity theory” ^46^ has rightly emphasized the temporal dimension of immune stimuli and responses. Although hotly debated about adaptative immunity ^47^, these issues have also be discussed in evolutionary contexts ^48^, and about innate immunity ^38^.

***Dynamic tuning.*** The concepts underlying « dynamic tuning », proposed in 1992 by Grossman and Paul ^49^, are best summarized by quotes taken from their 2015 review ^2^. As it does *“in other cell systems, neurons in particular, (…) dynamic tuning of cell responsiveness as the result of repeated stimuli, improves the ability of cells to distinguish physiologically meaningful signals from each other and from noise (…) eventhough the same sets of receptors may be utilized. In particular, lymphocyte activation thresholds are subject to tuning (…). Such tuning is also implicated in conferring flexibility to positive selection in the thymus, in controlling the magnitude of the immune response, and in generating memory cells. Additional functional properties are dynamically and differentially tuned in parallel via subthreshold contact interactions between developping or mature lymphocytes and self-antigen-presenting cells. (…). The built-in adjustability of intracellular control is utilized by the immune system to improve its organization and function”* ^50^. Importantly, each of these statements has received experimental support.

The three following points are most relevant for the current discussion:

(i) cells may exist in a variety of states, even with the same sets of receptors, and this feature is a built-in property of their intracellular network;

(ii) repeated stimulations help extracting meaningful signals out of environmental noise;

(iii) subthreshold contact interactions between two cells may re-frame phenotypes (thus, in the periphery, transplanted mature naive CD8+ or CD4+ T lymphocytes retain their phenotype -without being activated- in congruent MHC+ mice, but loose it in MHC knock out mice). This type of self-recognition has been shown to promote the foreign antigen sensitivity of naive T cells ^51^.

***The maximization of their diversity helps actors of immunity to defeat chance.*** This major lesson derives from the diversity of antibodies and TCRs and the size of their repertoires. A large number of antibodies are generated at random. Affinity maturation increases their diversity, then their specificity. The exchange of constant regions (isotype switching) adds to diversity by promoting topological and functional redistributions of antibody repertoires. TCRs are also generated at random in large numbers. There is no TCR maturation; instead, T cells tune their reactivity in order to optimize their avidity for their partner (or target).

Remarkably, immune responses can be elicited against just about anything. This holds for mice and men ^52^, and in even for mutant mice with restricted repertoires which have (at least) 100 to 1000 times fewer distinct antibodies, B cells and T cells than humans ^53^. Nevertheless, in all cases, the space of antigenic shapes is seemingly saturated. Therefore, adaptive responses are essentially scale free, implying that there is considerable potential for crossreactions between a given antibody or TCR and their molecular ligands ^40, 54^. This broad crossreactivity does not contradict the ‘exquisite’ specificity of the immune reactions, because specificity is raised to the appropriate level by additional mechanisms. As happens in T cells, tuning is a further means to broaden the diversity of phenotypes displayed by a given cell before adjusting its specificity.

### Extrapolated postulates

***All cells are auto-adaptable by tuning.*** I now postulate that *all* cells can adapt themselves and ajust their level of response according to their environment (this being a built-in property of all cellular networks). Grossman and Paul ^2^ quote evidence of tuning in B cells, NK cells, eosinophils and dendritic cells. Beyond, tuning would apply to all cells, whether associated with immunity (cf. the plasticity of macrophages ^55, 56^), or not (fibroblasts, liver cells, and others).

***Spontaneous and/or induced fluctuations are necessary to expand the diversity of NON-self recognition.*** Thus, in order to cope with chance, the natural defense system needs to maximize its diversity of interactions. It does so by various means, one being to exploit the fluctuations of intracellular networks. This assumption is supported by the broad dispersion of gene transcription and expression observed in isolated, genetically identical, cells in the same environment ^57– 59^. A population of cells is, therefore, dynamically much more diverse than one might expect, and its ability to sense the environment much broader. Subsequently, the adaptative character of tuning through repeated stimulations is essential to extract meaningful signals out of environmental noise, and deal with them appropriately. There is experimental evidence that a given T cell which gives rise to a disparate progeny makes up a more robust immunity ^60^. One may also hypothesize that tuning in innate cells helps understand why innate immunity alone protects many organisms so efficiently, despite the limited number of innate receptors.

***Cells check upon themselves.*** In an isolated cell, all pathways leading to apoptosis are linked to the detection of mistakes that could not be corrected to a sufficient degree, or of deviations of the internal network that could not be re-balanced. The cell is programmed in such a way that it then commits suicide. Apoptosis is, for the isolated cell, the ultimate correction mechanism.

***Cells double-check upon their neighbours.*** Cellular interactions are critical to maintain physiology, by ‘education’, selection or eradication. For example, in the periphery, subthreshold tuning based upon transient interactions with other cells is critical to maintain the properties of positively selected T lymphocytes. Apoptosis of sick cells triggered by normal ones is another type of check. Altogether, a moderately sick cell may correct itself, or be set back on track by a neighbour. If severely or irreversibly sick, it may commit suicide, or be killed by a neighbouring cell which induces its apoptosis or delivers lethal agents. Thus, cellular sickness is quality controlled from inside, and by neighbours.

###  The central role of cellular norms

Physiological norms concern the entire organism, but many lie at the cellular level. Intracellular norms mostly involve macromolecules, and translate into affinity constants (such as the affinity of a transcription factor for a promoter). The corresponding error detection and correction mechanisms pertain to the ‘civil defenses’. When by-passed, the cell becomes sick, and is taken care of by intra- and inter-cellular mechanisms. Higher scale disorders, in organs or the whole organism, are somehow referred back to individual cells (by lymphokines, hormones, metabolites, electric and other physical signals). Apart from extracellular microbes, the pathological objects to be discriminated are diseased cells (infected, tumoral or other). Thus, it makes sense to focus on cells and cellular norms.

### The normative self model

***The two types of auto-ajustable cells.*** (i) All cells undergo variations in their internal networks, and may, therefore, drift. However, those which are in constant contact with others in tissues and organs, are restricted in their capacity of tuning and cannot drift. If they did, they would die by apoptosis (either internal, or inflicted by their neighbours). This process is a basis of aging: tissues and organs are slowly empoverished, but not put in immediate danger, untill losses become too important (which stem cells may compensate). Cells belonging to tissues and organs constitute the « *somatic self* », which provides the necessary reference for the self-assessment of the physiological self. It follows the physiological variations of the organism over its life time.

(ii) In contrast, mobile cells are not under permanent mutual control. They are in a ‘relaxed tuning’ state, and are susceptible to drift away from their standards. They need to be ‘reset’ by periodic encounters with cells of the somatic self (for example, a circulating lymphocyte will meet a a blood vessel epithelial cell more frequently than another lymphocyte). Thus, mobile cells, are allowed to fluctuate, but within certain limits.

(iii) Both drift and reset may depend on the environment. The somatic self is likely not to be the same everywhere in the body and may differ in vessels, lymph nodes, or various tissues. For example, the same T cells may display distinct cytotoxic capabilities depending on their location, as suggested by the up-regulation of PD-1 in an allogeneic hematopoietic stem cell transplantation experiments ^61^.

**Figure 2.**
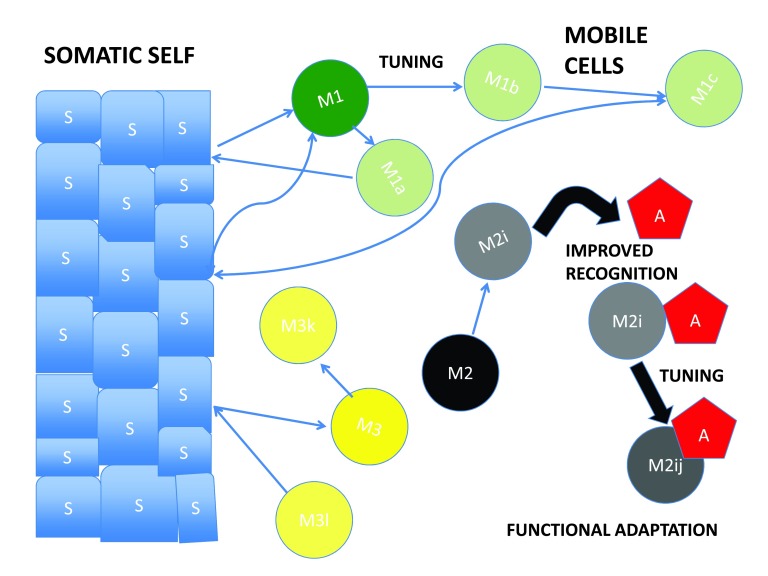
The normative self model The left side shows maximization of diversity of mobile cells (M) under the control of somatic cells (S). Thanks to tuning, various M cells (M1, M2, M3…) (in green, yellow and black) undergo diversification into (M1a, M1b…), (M2i, M2j …), (M3k, M3n…) (same colors with small variations), through deformations of their internal networks. S cells cannot diversify much because they are in close permanent contact. Diversified M cells (such as M1a, M3k, M3n) are occasionally reset to the M1 and M3 states by contact with S cells. The right side shows an aberrant cell (A) (in red), which is either altered (cancer) or infected. Thanks to network deformations (tuning), the population of mobile cells is more diverse, and there is a higher probability that a cell (M2i) properly recognizes the aberrant cell A. The recognition may be further improved by dynamic tuning which turns M2i into the more efficient M2ij, which will functionally adapt to deal with A.

***The professional army of the body is mostly recruited amongst mobile cells.*** The natural defense system thus combines civil defenses, disseminated in the entire body, with the services of a mobile professional army (which also has to defend itself, as any army does). Since hazards of life mostly happen by chance, the professional army relies on tuning to gain efficiency. However, it is constantly referred to the somatic self to keep in line with physiological standards ( Figure 2).

***Discrimination between the self and the NON-self.*** Discrimination is based on a simple principle: anything which lies off the norms is detected, corrected and/or destroyed, by civil defenses and/or the professional army. The fact that the latter needs ‘relaxed tuning’ to maximize its recognition capabilities creates a kind of no man’s land in the physiological self, a space where norms are less precise, and more opportunities given to crossreactions, mimicry, escape and so forth. Note that this grey zone may also be exploited by the NON-self. For example, tumor cells may use tuning to find their way out of host defenses. Infectious agents may do the same, thanks to so far unsuspected escape mechanisms.

## Discussion

### The natural defense system and the notion of robustness

The proposed ‘natural defense system’ exceeds by far the immune system. Just as the latter, it is a pure mental construction, which I deem worthy of consideration for several reasons:

***It unifies the fields of infectious diseases and internal pathologies.*** It provides a more comprehensive view of cancers, and facilitates the conceptual integration of immunology with other biological systems (for example, by linking microbiota and neurological diseases through inflammation).

***It emphasizes the so far underestimated role of quality control and error correction mechanisms.*** Admittedly, it may seem difficult and/or artificial, to distinguish an error correction device from the primary biological mechanism when both are part of the same macromolecular complex. For example, in DNA replication, it is easier to identify mismatch repair enzymes than the proof-reading activity of DNA polymerase. Nevertheless, quality control and error correction mechanisms should be more systematically investigated. Note that their occasional failure may be pathological. Thus, certain manifestations of auto-immune diseases may be due to defective quality control rather than to primary defects.

***The concept of robustness is fundamental.*** It provides a unified function to the natural defense system, which it links to evolutionary forces (its relationship with the concept of fitness would deserve attention).

***The natural defense system, which provides robustness to the organism, must itself be robust.*** Its peculiar architecture, as depicted in this paper, has been selected to be so. It will be interesting to examine whether it resembles certain engineered systems ^34^, and/or may inspire the design of new ones.

***The overall scheme is relatively easy to communicate and teach.*** This is not a minor point, as immunology is rightly reputed to be abstruse for students and for the general public.

### The natural defense system and physiology

The two definitions of the self *(the physiological organism at all stages of its life)* and of the natural defense system are consistently bridged by the notion of physiology. The physiological self, in its structural and functional dimensions, relies on critical interactions between proteins and cells, some of which may be expressed as affinity and avidity constants, thresholds and/or windows. This set of physiological standards is particularly important for mobile cells (such as T cells).

### The normative self model

The model is based on (a) physiological norms, and (b) their verification by an adequately structured control and defense system.

***Physiological norms*** are somehow written in the genome. Their implementation during development, as well as their maintenance during life, rely on the body plans of the natural defense system, which should be further explored ^62^. The set of norms must itself be robust with respect to the numerous genetic and epigenetic changes which occur in the body. In humans, the rate of somatic mutations (10 ^-8^/10 ^-9^) and the number of cell divisions from germ line to adult (by the hundreds for spermatozoa) are high enough to generate many gametes bearing multiple mutations. The latter should be viewed as impacting their internal networks, rather than single genes or simple functions. A network adapted genetic thinking is thus needed. I have earlier speculated that the ‘domestic’ part of body plans may undergo selection at checkpoints during gametogenesis ^63^.

***The verification of norms*** is the duty of an army which relies on two categories of cells: the non mobile, aggregated ones, which make up the somatic self (where most civil defenses lie), and the mobile ones (which make up the core of the professional army). The latter have to periodically refer to the somatic self to keep in line with the physiological standards.

***Comments*** - The tuning-based extension of recognition capabilities of mobile cells might generate uncontrolled drift, and the natural defense system would not be robust enough, if mobile cells were not checked by immobile cells. This also provides a means by which the set of mobile cells adapts to age.

- The generalization to all cell types of the notion *of tuning,* initially elaborated for T lymphocytes, might be further documented by analyzing the different states of intracellular networks in single cells of various types. The ‘elasticity’ of cellular networks (a particular kind of epigenetic phenomenon) might be modeled by introducing the notion of *entropy* at the single cell level, following Shannon’s theory of information ^64, 65^.

- Current technologies ^66, 67^ allow to study the *interactions of a single mobile cell with an immobile layer of cells*, mimicking a tissue. The model predicts a change in the internal networks distribution of the single cell (a reduction of the cellular entropy).

- It would be important to *define the physiological borders of the relaxed tuning state.* One may speculate that the internal networks of cells in a relaxed tuning state display an elastic behaviour, which would periodically drive them back to average. Even so, it would be surprising that the borders are not surveyed at certain checkpoints involving: Fas and Fas ligand (for apoptosis), MHC expression (for NK cells), cell surface markers such as CD59 (as inhibitors of complement), and possibly others as yet undiscovered.

- It will be critical to evaluate ( *in vitro*, and preferably *in vivo*) the *time which elapses between two effective checks*. This parameter is key, because it defines the time window during which interventions can be made. Repeating interventions at proper times may thus lead to *desensitization*, as already recognized and discussed ^2, 45^.

- Immune changes with time, (particularly aging), have been mostly analyzed at the level of the components of the immune system. Additional studies might relate changes in natural defenses with the physiological evolution of the somatic self. This may help understand pathological variations in relation with age.

### A dynamic view of physiology

These above considerations support a very dynamic view of physiology, which results from the realization that, in Claude Bernard’s *milieu intérieur*, the elements are themselves uncertain, and, to some extent, renewable. Current research promotes a vision of our body that evokes the apologue of the Delphies’ boat, which seemingly remains the same, while most of its constituents have been changed. Our body does so by itself (though now complemented by medicine), with 70 billions cells renewed every day.

We are largely unaware of these uncessant internal fights and of the remarkable robustness of our organism. It may be more comfortable to ignore that our natural defense system permanently prevents and/or cures many infections, cancers, cardiovascular disorders, and so on. Nevertheless, understanding, then mastering better, these physiological dynamics, which maintain a stability slowly destroyed by physiological aging, will ultimately help improve our health.
