# Supplementary material for: The natural defense system and the normative self model
Source: F1000Res. 2016 May 3;5:797. [Version 1] doi: 10.12688/f1000research.8518.1 (PMC4890299; doi:10.12688/f1000research.8518.1)
Supplement: Peer review history

# Peer review: Referee response for version 1

**DOI:** 10.5256/f1000research.9169.r13692

**Published:** 2016-05-27

**Authors:** David H. Margulies, Kannan Natarajan

**Affiliations:** 1 Molecular Biology Section, Laboratory of Immunology, National Institute of Allergy and Infectious Diseases, National Institutes of Health, Bethesda, MD, USA; 2 Molecular Biology Section, Laboratory of Immunology,, National Institute of Allergy and Infectious Disease, National Institutes of Health, Bethesda, MD, USA

In this opinion article, Kourilsky articulates a sweeping, holistic perspective on the plethora of innate defense mechanisms that exist at the molecular, cellular, tissue, organ, and organismal levels to ensure survival. The author broadens the definition of innate defenses to include not only cells traditionally associated with immunity such as macrophages and dendritic cells, but rather each and every cell and organ system in the organism.

 The division of function is between cells that are sessile, such as those organized into tissues and organs, and that are motile such as those of the hematopoetic system. A key idea is the “normative self” – a homeostatic physiological state to which both types of cells contribute. The normative self is learned and is continuously reinforced by interactions between sessile and motile cells that tune the system to respond appropriately to neoplastic cells, aberrant inflammatory responses, and environmental perturbations such as infectious disease. The essay is thought-provoking and attempts, successfully in our opinion, to highlight the landscape of adaptable interactions that is necessary to sustain life. Conceivably, this perspective may be applied beyond the individual organism to the interplay of communities and even higher order populations.

We have read this submission. We believe that we have an appropriate level of expertise to confirm that it is of an acceptable scientific standard.

# Peer review: Referee response for version 1

**DOI:** 10.5256/f1000research.9169.r14013

**Published:** 2016-05-26

**Authors:** Bernard Malissen

**Affiliations:** 1 Centre d'Immunologie de Marseille-Luminy, Aix-Marseille University UM2, Inserm, U1104, CNRS 10, UMR7280, Marseille, France

The opinion article by P. Kourilsky proposes a new model of the immune system called the «normative self model». It is vividly written and discusses several former models - such as the one promoted by Paul and Grossman - and it also capitalizes on recent experimental findings. It will be interesting to add a couple of sentences on innate lymphoid cells, unconventional T cells (e.g. MAIT, gd T cells, and CD8aa+ IELs), and tissue-resident memory T (TRM) cells since these diverse cell types play a role in the preservation of tissue integrity and function during homeostasis, infection, and non-infectious perturbations. More importantly, the aim of a model is to provide experiments that can be tested and clearly refuted or validated. Therefore, I will strongly encourage P. Kourilsky to include a section in which a couple of such experiments are suggested.

I have read this submission. I believe that I have an appropriate level of expertise to confirm that it is of an acceptable scientific standard.
